# DIVERT-Ca: unveiling the hidden link between acute diverticulitis and colorectal cancer risk—multicentre retrospective study

**DOI:** 10.1007/s00384-025-04858-1

**Published:** 2025-03-15

**Authors:** Mohamed Talaat Issa, Emiko Sultana, Mohammed Hamid, Ali Yasen Mohamedahmed, Mohamed Albendary, Shafquat Zaman, Santosh Bhandari, William Ball, Sangara Narayanasamy, Pradeep Thomas, Najam Husain, Rajeev Peravali, Diwakar Sarma

**Affiliations:** 1 Department of General and Colorectal Surgery, Sandwell and West Birmingham NHS Trust, Birmingham, UK; 2 Department of General and Colorectal Surgery, Shrewsbury and Telford Hospitals NHS Trust, Shrewsbury, UK; 3 Department of General and Colorectal Surgery, Walsall Manor Hospital, Walsall Healthcare NHS Trust, Walsall, UK; 4 Department of General and Colorectal Surgery, University Hospitals of Derby and Burton NHS Foundation Trust, Queen’s Hospital Burton, Burton On Trent, UK; 5 Department of General Surgery, North West Anglia NHS Trust, Peterborough, UK; 6 College of Medical and Dental Sciences, School of Medicine, University of Birmingham, Edgbaston, Birmingham, UK

**Keywords:** Colorectal cancer, Diverticulitis, Endoscopy, Colonoscopy

## Abstract

**Introduction:**

Colorectal cancer (CRC) is the third most common cancer worldwide, accounting for approximately 10% of all malignancies. Emerging trends of association with risk factors such as diverticulitis highlight the need for updated screening and follow-up protocols. We aimed to examine risk factors associated with the development of CRC within 12 months following an episode of acute diverticulitis, and identify areas to streamline follow-up.

**Methods:**

We performed a retrospective multicentre study of adult patients admitted in 2022 with computed tomography (CT) confirmed acute diverticulitis across four large NHS Trusts in the UK. Patient demographics, comorbidities, clinical presentation, vital signs, laboratory results, details of in-patient stay, and follow-up investigations were collected and analysed. Our primary outcome was the incidence of CRC within 12 months of index presentation with acute diverticulitis. Analysed secondary outcomes were potential patient risk factors associated with a diagnosis of CRC and follow-up protocols. All statistical analysis was performed using R (version 4.4) and *P*-values of < 0.05 were considered statistically significant.

**Results:**

A total of 542 patients with acute diverticulitis over the study period were included. The median age of our cohort was 62 (51–73) years, and 204 (37.6%) were male. Ten (1.8%) patients were diagnosed with CRC within the 12-month period. Hinchey grade Ib was significantly associated with CRC (OR 4.51, *P* = 0.028). Colonoscopic follow-up requests were associated with age between 40 and 60 years, mild white cell count (WCC) elevation, and a hospital stay of 3–7 days. Male gender, age between 18 and 40 years, and elevated C-reactive protein (CRP) were all strongly associated with CRC but not statistically significant. Follow-up was inconsistent with 53.7% of the cohort having luminal investigations.

**Conclusion:**

The incidence of CRC was in-keeping with published literature. Hinchey grade 1b was significantly associated with a subsequent CRC diagnosis. These findings emphasise the need for specialised radiological review of CT scans to detect underlying malignancy. Moreover, standardised follow-up protocols following an episode of acute diverticulitis are needed to avoid missing malignant lesions.

**Supplementary Information:**

The online version contains supplementary material available at 10.1007/s00384-025-04858-1.

## Introduction

Colorectal cancer (CRC) accounts for approximately 10% of the total cancer burden worldwide, making it the third most common cancer and the second leading cause of cancer-related mortality [1]. Studies have suggested that the incidence and mortality associated with CRC can be significantly reduced through primary, secondary, and tertiary prevention. These measures include identifying risk factors/high-risk groups, implementing effective screening programmes, promoting healthy lifestyle choices, and increasing awareness, early diagnosis, and treatment [2–4].

Risk factors associated with the development of CRC include advanced age, significant family history, high-risk colonic polyps, sedentary lifestyle, smoking and alcohol consumption, and the consumption of processed meat [1, 2, 5–8]. However, with ever-changing global trends and patterns, there is an emergent need to re-consider the association between other risk factors and the development of bowel cancer and by extension our current screening strategies.

Interestingly, the age distribution of patients presenting with CRC has changed considerably over the past decade. CRC cases diagnosed before the age of 50 years have continued to increase, whilst the incidence in those above 50 years has reduced [9, 10]. These findings would warrant a lowering of the age for entering bowel cancer screening from 50 years to 40 (UK).

Furthermore, a strong association between acute diverticulitis and the prevalence of CRC has been suggested [11, 12]. Due to the risk between diverticular disease (DD) and CRC occurrence, the Association of Coloproctology of Great Britain and Ireland (ACPGBI) and the American Gastroenterological Association (AGA) both advocated for performing endoscopic investigations (colonoscopy) in patients diagnosed with acute diverticulitis [13, 14].

These are usually performed 6–8 weeks following the acute flare-up of disease or until complete resolution of symptoms [13]. Patients with complicated grades of diverticulitis are on average six times more likely to have a diagnosis of CRC compared with uncomplicated cases [15, 16].

Recent recommendations suggest that colonoscopies should be reserved for patients presenting with complicated diverticulitis [17, 18]. ACPGBI (2022) guidelines state that in-patients managed non-operatively in instances of acute uncomplicated diverticulitis, follow-up colonoscopic or radiological investigations are not required unless the presence of suspicious CT features or other risk factors for malignancy indicate otherwise.

Colonoscopy is recommended in all patients following an attack of complicated acute diverticulitis after 6 weeks (unless imaging has been performed in the preceding 2 years) [19]. This is because acute diverticulitis is increasingly being diagnosed with the aid of CT scans, providing high-resolution images, instead of relying solely on clinical diagnosis and acumen [18, 20]. In the majority of cases, diverticulitis and CRC can be accurately determined, but CT is unable to exclude neoplasia in approximately 10% of patients [21–23].

Historically, there has been a higher incidence of left-sided CRC (L-CRC) compared with right-sided lesions [24]. Studies have demonstrated that patients with L-CRC have chromosomal instability with pathway-related mutations, such as KRAS (Kirsten rat sarcoma virus), APC (adenomatous polyposis coli), PIK3CA (phosphatidylinositol-4,5-bisphosphate 3-kinase catalytic subunit alpha), and p53 mutations.

In contrast, patients with right-sided colon cancer (RCC) have DNA mismatch repair pathway mutations. Therefore, patients with L-CRC generally benefit from adjuvant chemotherapy, and patients with RCC are showing promising results with immunotherapies but do not respond well to conventional chemotherapy [25, 26].

Despite the possible association between DD and CRC, the key predicting factors that predispose individuals with diverticulitis to CRC remain unclear. We aimed to determine the incidence and patient factors associated with a diagnosis of CRC 12 months following an admission with acute diverticulitis, and explore follow-up in such patients.

## Methods

### Study design and setting

We performed a retrospective multicentre study across four large hospitals in the UK (Peterborough City Hospital, Queen’s Hospital Burton, Sandwell General Hospital, and Royal Shrewsbury Hospital). The local audit department of each participating hospital approved the study which was conducted and reported in accordance with the Declaration of Helsinki and STROBE recommendations (Appendix 1). All patients presenting with CT-confirmed acute diverticulitis from 01/01/2022 till 31/12/2022 were considered against the study inclusion and exclusion criteria. All included patients had a follow-up period of at least 12 months.

### Patient selection criteria

Electronic medical records were searched to identify eligible cases at each hospital site. Our inclusion criteria were adult patients (aged ≥ 18 years) presenting with abdominal pain and a CT scan showing acute colonic diverticulitis. The modified Hinchey classification system was used to grade the severity of diverticulitis [27]. Uncomplicated diverticulitis is defined by the lack of associated complications (Hinchey Ia), whereas complicated diverticulitis is associated with the formation of abscesses, fistula, bowel obstruction, or frank perforation (Hinchey Ib-IV).

Hinchey grade Ia was defined as confined/localised pericolic inflammation (phlegmon); grade Ib for confined pericolic abscess; grade II for pelvic, intraabdominal, or retroperitoneal abscess; grade III for generalised purulent peritonitis; and grade IV for faecal peritonitis.

Patients with diverticulitis in the gastrointestinal tract other than the colon and patients diagnosed clinically without radiological confirmation (no CT scan) or with equivocal CT findings were excluded. Incomplete datasets also precluded patients from the study.

### Data collection

Data were extracted for all eligible patients from electronic and archived records, and stored on an encrypted, password-protected computer. Data included demographics (age, gender, comorbidities (including previous episodes of diverticulitis), and steroid use), vital signs scores (temperature (°C)), respiratory rate, blood pressure (mmHg), and levels of consciousness assessed using the Glasgow Coma Scale (GCS), inflammatory markers on presentation (White Blood Cell Count (WCC, × 10*9/L) and C-reactive protein (CRP, mg/L), antibiotic prescription, treatment setting (ambulatory vs. in-patient), length of hospital stay (LOS), and follow-up colonic investigation(s).

All patients were followed up for 12 months after the index presentation, and the incidence of CRC diagnosis was recorded. In this study, patients staying in hospital for ≥ 24 h or those requiring intensive therapy unit (ITU) support were categorised as in-patients. Ambulatory care was defined as < 24-h hospital stay.

### Outcomes

Our primary outcome of interest was the incidence of CRC diagnosed within 12 months of the index presentation with acute diverticulitis. Analysed secondary outcomes were patient factors and index presentation findings associated with a subsequent diagnosis of CRC. Follow-up of these patients and further investigations requested were also explored.

### Statistical analysis

The Kolmogorov-Smirnov test of normality demonstrated a D-statistic of 0.050, *P* = 0.003. The data were summarised using median and inter-quartile range (IQR) for continuous variables and number and percentage for categorical data. Odds ratio (OR) and 95% confidence intervals (95% CI) were calculated for patient factors and index presentation findings associated with a CRC diagnosis within 12 months of presenting with acute diverticulitis, and for follow-up and investigations requested, using both univariate and multivariate binomial logistic regression (adjusting for age, gender, and comorbidities).

*P*-values of < 0.05 were considered statistically significant. All statistical analysis was performed using R version 4.4 (R Foundation for Statistical Computing, Vienna, Austria).

## Results

### Patient characteristics

A total of 542 patients diagnosed with acute diverticulitis during the study period were included. The median age of our cohort was 62 (51–73) years, and 204 (37.6%) were male. Patient characteristics are summarised in Table 1.

**Table 1:** Study patient characteristics

Characteristic	All (*n* = 542)
Age, median (IQR) years	62 (51–73)
18 – < 40	39 (7.2)
40 – < 60	212 (39.1)
60 – < 80	220 (40.6)
≥ 80	71 (13.1)
Male gender, *n* (%)	204 (37.6)
Diabetic, *n* (%)	54 (10.0)
Use of steroids, *n* (%)	27 (5.0)
Previous diverticulitis, *n* (%)	174 (32.1)
Complicated	57 (10.5)
Vital signs, *n* (%)	
Temperature ≥ 38 °C	36 (6.6)
Elevated RR ≥ 20	19 (3.5)
Low SBP ≤ 90 mmHg	33 (6.1)
Altered GCS < 15	5 (0.9)
Inflammatory markers, *n* (%)	
WCC (× 10*9/L)	
< 10	163 (30.1)
10–15	231 (42.6)
15–20	112 (20.7)
> 20	36 (6.6)
CRP (mg/L)	
< 50	195 (36.0)
50–100	114 (21.0)
100–200	166 (30.6)
200–300	47 (8.7)
> 300	20 (4.0)
Hinchey grading, *n* (%)	
1a	348 (64.2)
1b	94 (17.3)
2	54 (10.0)
3	36 (6.6)
4	10 (1.8)
Antibiotics prescription *n* (%)	526 (97.0)
Ambulatory care, *n* (%)	92 (17.0)
ITU admission, *n* (%)	17 (3.1)
Length of stay, median (IQR) days	3 (1–5)
Follow-up requested, *n* (%)	291 (53.7)
Endoscopy, *n* (%)	274 (50.6)
Weeks post-review, median (IQR)	7 (6–12)
Flexible sigmoidoscopy, *n* (%)	137 (25.3)
Colonoscopy, n (%)	137 (25.3)
CTC, *n* (%)	34 (6.3)
CRC within 1 year, *n* (%)	10 (1.8)

*IQR* inter-quartile range, *°C* degrees Celsius, *RR* respiratory rate, *SBP* systolic blood pressure, *GCS* Glasgow Coma Scale, *WCC* white cell count, *CRP* C-reactive protein, *ITU* intensive therapy unit, *CRC* colorectal cancer, *CTC* computed tomography colonography

Approximately one-third of patients (174, 32.1%) had a previous diagnosis of diverticulitis with 57 (10.5%) categorised as complicated presentations. Fifty-four (10.0%) were comorbid with diabetes mellitus, and 27 (5.0%) were on long-term steroids.

On initial hospital assessment, 36 (6.6%) had a fever ≥ 38 °C, 19 (3.5%) were tachypnoeic with a RR ≥ 20 breaths/min, 33 (6.1%) were hypotensive with a systolic blood pressure ≤ 90 mmHg, and five patients (0.9%) had altered consciousness with a GCS score of < 15.

Mild grades of diverticulitis were more common; 348 (64.2%) categorised as Hinchey grade Ia, 94 (17.3%) Hinchey 1b, 54 (10.0%) Hinchey II, 36 (6.6%) Hinchey III, and only 10 (1.8%) patients with Hinchey 4. The inflammatory markers linked to the presentations are summarised in Table 1.

Almost all 526/542 (97.0%) patients were prescribed antibacterial therapy. The majority of patients were treated as in-patients with 17 (3.1%) needing ITU admission. The median length of hospital stay was 3 days. A small proportion of patients (92, 17.0%) were managed in an ambulatory care setting.

### Colorectal cancer

In our cohort, 10 (1.8%) patients were diagnosed with CRC within 12 months of their acute diverticulitis presentation (characteristics of these patients are summarised in Table 2). Their age ranged between 41 and 87 years, 7 were male, 2 diabetics, and 4 had a previous diagnosis of uncomplicated diverticulitis. None of these patients was on long-term steroids. One patient had a previous breast cancer diagnosis and one had a first-degree relative affected by CRC.

**Table 2:** Characteristics of patients diagnosed with colon cancer within 1 year of index admission

	Patient number									
Characteristics	1	2	3	4	5	6	7	8	9	10
Age (years)	52	70	64	41	69	87	44	59	84	71
Male	-	Yes	Yes	-	Yes	-	Yes	Yes	Yes	Yes
Diabetic	-	-	Yes	-	-	-	-	Yes	-	-
Steroid use	-	-	-	-	-	-	-	-	-	-
Previous diverticulitis	-	Yes	-	-	-	-	-	Yes	Yes	Yes
Complicated	-	-	-	-	-	-	-	-	-	-
Previous cancer	-	-	-	-	Yes Breast	-	-	-	-	-
CRC in 1st degree relative	-	-	Yes	-	-	-	-	-	-	-
Index admission findings										
Presenting complaint	CBH	LIF pain	CBH	CBH	LIF pain	LIF pain	RUQ pain	CBH	LIF pain	LIF pain
Temperature ≥ 38 °C	Yes	-	-	-	-	-	-	-	-	-
Elevated RR ≥ 20	-	-	-	-	-	-	-	-	-	-
Low SBP ≤ 90 mmHg	Yes	-	-	-	-	-	-	-	-	-
Altered GCS < 15	-	-	-	-	-	-	-	-	-	-
WCC < 15 (× 10*9/L)	-	-	Yes	Yes	Yes	Yes	Yes	Yes	-	-
CRP > 100 (mg/L)	-	Yes	-	-	Yes	Yes	Yes	-	Yes	Yes
Hinchey grading	Ib	Ib	Ia	Ia	Ib	III	Ia	Ia	Ib	Ib
Antibiotics prescribed	Yes	Yes	Yes	Yes	Yes	Yes	Yes	Yes	Yes	Yes
Ambulatory care	-	-	-	Yes	-	-	-	-	-	-
ITU admission	-	-	-	-	-	-	-	-	-	-
Length of stay (days)	5	3	1	0	1	8	1	3	3	3
Follow-up requested	Yes	Yes	-	Yes	-	Yes	Yes	-	Yes	Yes
Endoscopy	Yes	Yes		Yes		Yes	Yes		Yes	Yes
Weeks from discharge	6	6	-	7	-	-	2	-	27	6
Flexible sigmoidoscopy	Yes	-	-	Yes	-	-	-	-	-	Yes
Colonoscopy	-	Yes	-	-	-	Yes	Yes	-	Yes	-
CTC	-	-	-	-	-	-	-	-	Yes	-
Location of CRC (R/L/T)	L	L	T	L	T	L	T	L	R	L
Diverticulitis admission to cancer diagnosis (days)	48	78	20	45	36	94	52	87	30	78
T stage	T4	T4	T1	T1	T3	T1	T3	T1	T4	T4
N stage	0	0	0	0	1	0	0	0	1	0
M stage	0	0	1 Liver	0	0	0	0	0	0	0
Cancer management (P/C)	P	C	P	C	C	P	C	C	C	C

*CRC* colorectal cancer, *CBH* change in bowel habit, *LIF* left iliac fossa, *RUQ* right upper quadrant, *°C* degrees Celsius, *RR* respiratory rate, *SBP* systolic blood pressure, *GCS* Glasgow Coma Scale, *WCC* white cell count, *CRP* C-reactive protein, *ITU* intensive therapy unit, *CTC* computed tomography colonography, *R* right, *L* left, *T* transverse (location), *T* tumour, *N* node, *M* metastasis (stage), *P* palliative, *C* curative

Half of these patients on presentation had left iliac fossa (LIF) pain, whilst four complained mainly of altered bowel habit and one described upper abdominal pain/discomfort. A single patient from this group displayed unstable vital signs (pyrexia and hypotension).

Inflammatory markers mainly showed a WCC < 15 × 10*9/L and CRP ranging between 100 and 200 mg/L. All patients were treated with antibiotics, one ambulatory and the remainder as inpatients, with hospital stay ranging between 1 and 8 days.

Seven of the ten patients had follow-up organised with a range of 2–27 weeks. Three patients had a flexible sigmoidoscopy and three colonoscopy. One patient had a CT colonography (CTC) followed by conventional colonoscopy. The remaining patients were diagnosed through a later CT scan.

The interval between index diverticulitis presentation and CRC diagnosis ranged between 20 and 94 days (median 50 days). The location of CRC was transverse colon (three patients), right colon (one), and the remainder in the left colon. Four patients had T4 disease, and one had metastasis. Seven patients were managed with curative intent and three palliated.

On multivariate analysis of patient factors (Table 3), Hinchey grade Ib was the only significant indicator for a CRC diagnosis within 1 year (OR 4.51 (1.23, 16.64), *P* = 0.028). Other strongly associated (but non-statistically significant) factors associated with a CRC diagnosis were age 18–40 years (OR 3.38, *P* = 0.160), male gender (OR 3.62, *P* = 0.057), CRP levels between 100 and 200 mg/L (OR 3.31, *P* = 0.069), and previous history of uncomplicated diverticulitis (OR 3.08, *P* = 0.112).

**Table 3:** Patient factors and index presentation findings associated with a colonic cancer diagnosis within 1 year using univariate and multivariate binomial logistic regression modelling

Characteristic	Univariate OR (95% CI)	*p*-value	Multivariate OR (95% CI)	*p*-value
Patient factors				
Age (years)	0.97 (0.93, 1.01)	0.169	0.97 (0.93, 1.01)	0.158
18– < 40	3.22 (0.66, 15.73)	0.119	3.38 (0.71, 20.77)	0.160
40– < 60	1.03 (0.29, 3.69)	0.939	0.95 (0.26, 3.46)	0.939
60– < 80	0.63 (0.16, 2.45)	0.550	0.66 (0.17, 2.61)	0.540
≥ 80	0.75 (0.09, 6.05)	0.786	0.74 (0.09, 6.26)	0.778
Male	3.91 (1.00, 15.31)	0.070	3.62 (0.90, 14.55)	0.057
Diabetic	2.43 (0.50, 11.76)	0.154	3.33 (0.64, 17.36)	0.197
Steroid use	0.00 (0.00, Inf)	0.991	0.00 (0.00, Inf)	0.418
Previous diverticulitis	1.40 (0.39, 5.02)	0.407	1.73 (0.47, 6.38)	0.417
Complicated	0.00 (0.00, Inf)	0.994	0.00 (0.00, Inf)	0.155
Uncomplicated	2.48 (0.69, 8.93)	0.097	3.08 (0.82, 11.65)	0.112
Index findings				
Temperature ≥ 38 °C	1.52 (0.19, 12.35)	0.883	1.18 (0.14, 10.11)	0.885
Elevated RR	0.00 (0.00, Inf)	0.995	0.00 (0.00, Inf)	0.431
Low SBP	1.79 (0.22, 14.62)	0.410	2.48 (0.29, 21.36)	0.457
Altered GCS	0.00 (0.00, Inf)	0.998	0.00 (0.00, Inf)	0.807
Inflammatory Markers				
WCC < 10	0.00 (0.00, Inf)	0.990	0.00 (0.00, Inf)	0.800
WCC 10–15	2.02 (0.56, 7.25)	0.231	2.21 (0.60, 8.08)	0.225
WCC 15–20	2.69 (0.75, 9.72)	0.186	2.44 (0.65, 9.17)	0.202
WCC > 20	0.00 (0.00, Inf)	0.993	0.00 (0.00, Inf)	0.216
CRP < 50	0.43 (0.09, 2.06)	0.352	0.47 (0.10, 2.30)	0.322
CRP 50–100	0.40 (0.05, 3.22)	0.400	0.41 (0.05, 3.32)	0.348
CRP 100–200	3.47 (0.97, 12.48)	0.072	3.31 (0.90, 12.16)	0.069
CRP 200–300	1.25 (0.015, 10.09)	0.917	1.12 (0.13, 9.33)	0.918
CRP > 300	0.00 (0.00, Inf)	0.995	0.00 (0.00, Inf)	0.387
Hinchey grading				
1a	0.37 (0.10, 1.31)	0.149	0.38 (0.10, 1.41)	0.144
1b	4.91 (1.39, 17.32)	0.023*	4.51 (1.23. 16.64)	0.028*
2	0.00 (0.00, Inf)	0.994	0.00 (0.00, Inf)	0.189
3	1.73 (0.21, 14.10)	0.814	1.29 (0.15, 11.13)	0.819
4	0.00 (0.00, Inf)	0.996	0.00 (0.00, Inf)	0.563

^*^Statistically significant

*95% CI* 95% confidence interval, *RR* respiratory rate, *SBP* systolic blood pressure, *GCS* Glasgow Coma Scale, *WCC* white cell count, *CRP* C-reactive protein

### Colonic investigations

A total of 291 (53.7%) patients had follow-up requested with a median time of 7 (IQR 6–12) weeks post admission. Of these, flexible sigmoidoscopy was performed in 137 (25.3%) patients and an equal number had colonoscopy (25.3%). The remainder had a CTC.

On multivariate analysis of patient factors (Table 4), age group 40–60 years (OR 1.70, *P* = 0.003), WCC 10–15 × 10*9/L (OR 1.51, *P* = 0.021), and length of hospital stay of 3–7 days (OR 1.90, *P* < 0.001) were all significantly associated with a request for colonic follow-up. Patients over the age of 80 years were less likely to be investigated further (OR 0.53, *P* = 0.016).

**Table 4:** Salient and significant patient factors and index presentation findings associated with follow-up and colonic investigation using univariate and multivariate binomial logistic regression modelling

Characteristic	Univariate OR (95% CI)	*p*-value	Multivariate OR (95% CI)	*p*-value
Follow-up requested				
Age 18–40	0.81 (0.42, 1.55)	0.286	0.70 (0.36, 1.35)	0.286
Age 40–60	1.77 (1.25, 2.52)	0.004*	1.70 (1.19, 2.42)	0.003*
Age > 80	0.51 (0.31, 0.86)	0.017*	0.53 (0.32, 0.89)	0.016*
Male	1.12 (0.79, 1.58)	0.866	1.03 (0.72, 1.47)	0.866
PDD—uncomplicated	0.90 (0.60, 1.36)	0.835	0.96 (0.63, 1.46)	0.835
WCC 10–15	1.53 (1.09, 2.16)	0.021*	1.51 (1.06, 2.13)	0.021*
CRP 100–200	1.03 (0.71, 1.49)	0.997	1.00 (0.69, 1.45)	0.997
Hinchey grade Ib	1.14 (0.73, 1.79)	0.738	1.08 (0.68, 1.71)	0.738
LOS 1 day	0.73 (0.46, 1.15)	0.068	0.64 (0.40, 1.03)	0.067
LOS 2 days	0.88 (0.56, 1.40)	0.481	0.85 (0.53, 1.53)	0.482
LOS 3–7 days	1.80 (1.24, 2.60)	< 0.001*	1.90 (1.30, 2.78)	< 0.001*
LOS < 7 days	0.61 (0.38, 0.98)	0.092	0.66 (0.40, 1.07)	0.091
Flexible sigmoidoscopy				
Age 18–40	1.18 (0.57, 2.43)	0.799	1.10 (0.53, 2.30)	0.800
Male	1.15 (0.77, 1.71)	0.542	1.13 (0.76, 1.69)	0.543
PDD—uncomplicated	0.93 (0.57, 1.49)	0.886	0.96 (0.59, 1.57)	0.886
CRP 100–200	1.15 (0.76, 1.74)	0.502	1.15 (0.76, 1.75)	0.504
CRP > 300	0.32 (0.07, 1.39)	0.339	0.33 (0.07, 1.42)	0.086
Hinchey Ib	1.40 (0.86, 2.29)	0.235	1.35 (0.82, 2.22)	0.241
Hinchey grade 3	0.46 (0.17, 1.20)	0.104	0.45 (0.17, 1.18)	0.077
Colonoscopy				
Age 18–40	0.75 (0.34, 1.67)	0.280	0.64 (0.28, 1.44)	0.263
Age 40–60	1.86 (1.23, 2.75)	0.005*	1.76 (1.19, 2.63)	0.005*
Age > 80	0.34 (0.16, 0.72)	0.006*	0.34 (0.16, 0.73)	0.002*
Male	1.25 (0.84, 1.86)	0.562	1.13 (0.75, 1.69)	0.563
Steroid use	0.23 (0.05, 0.96)	0.070	0.26 (0.06, 1.12)	0.030*
Previous diverticulitis	0.59 (0.38, 0.92)	0.057	0.65 (0.41, 1.01)	0.053
PDD—uncomplicated	0.63 (0.38, 1.06)	0.143	0.68 (0.40, 1.14)	0.135
CRP 100–200	0.87 (0.57, 1.33)	0.400	0.83 (0.54, 1.28)	0.397
Hinchey Ib	0.95 (0.57, 1.59)	0.646	0.88 (0.52, 1.50)	0.644
Reduced hospital stay	0.95 (0.91, 1.00)	0.077	0.96 (0.92, 1.00)	0.049*
LOS 1 day	0.70 (0.40, 1.23)	0.081	0.60 (0.34, 1.06)	0.072
LOS 3–7 days	1.35 (0.88, 2.05)	0.122	1.40 (0.91, 2.16)	0.123
CT colonography				
Age > 80	0.63 (0.19, 2.10)	0.488	0.65 (0.19, 2.20)	0.465
LOS 2 days	0.30 (0.07, 1.27)	0.092	0.29 (0.07, 1.23)	0.046*

^*^Statistically significant; *95% CI* 95% confidence interval, *PDD* previous diverticulitis diagnosis, *RR* respiratory rate, *SBP* systolic blood pressure, *GCS* Glasgow Coma Scale, *WCC* white cell count, *CRP* C-reactive protein, *LOS* length of stay

This pattern was similar for colonoscopy requests, but not for flexible sigmoidoscopy which did not demonstrate any particular pattern. Table 4 also shows the odds of a colonic follow-up and investigation request for patient factors strongly associated with a CRC diagnosis at one year (Hinchey Ib, age 18–40 years, male gender, CRP 100–200 mg/L, and previous uncomplicated diverticulitis), as described above. The odds of requesting colonic follow-up investigations for these factors were non-significant.

## Discussion

The present study demonstrates an incidence of 1.8% CRC diagnosis within 1 year of admission with episode of acute diverticulitis in-keeping with published literature (0.7–2% reported risk) [28]. This emphasises the need to be alert to the possibility of CRC in patients presenting with acute diverticulitis, as symptoms and radiological appearances may overlap rendering it difficult to differentiate malignancy from inflammatory changes [22].

### Key predictors of CRC diagnosis post-diverticulitis

In our cohort, Hinchey grade Ib diverticulitis (OR 4.51, *P* = 0.028) was strongly associated with CRC risk. This could be explained by the presence of localised inflammation/para-colonic collections making it difficult to detect mucosal thickening or the presence of small tumours on CT scan [28, 29].

High-resolution imaging techniques and/or reviewing acute diverticulitis scans with a specialised gastrointestinal radiologist may be warranted in helping to identify early malignant disease [30]. We also noted a strong (but statistically non-significant) association between previous uncomplicated diverticulitis and mild-moderately raised inflammatory markers (CRP 100–200 mg/L) with a subsequent CRC diagnosis (OR 3.08, *P* = 0.112) and (OR 3.31, *P* = 0.069) respectively. These findings are supported by previous studies linking chronic inflammation with carcinogenesis [31].

Male gender (OR 3.62, *P* = 0.057) and patients aged 18–40 years (OR 3.38, *P* = 0.16) showed a strong likelihood of a subsequent CRC diagnosis in line with recent observed epidemiological trends. Recent increases in CRC detection rates in patients younger than 50 years of age have been attributed to environmental, genetic, and lifestyle factors [32]. In males, hormonal, sex-related, and healthcare-seeking behavioural differences have been postulated as possible drivers for this phenomenon [33].

### Left- vs. right-sided CRC

L-CRC is more prevalent amongst western populations [34], and this was also evident in our cohort. In our study, L-CRC represented 70% of all cases, with transverse colon (20%) and right colon (10%) location making up the rest. L-CRC presents with varying symptoms including altered bowel habit and bleeding per rectum. Diagnostic difficulties are encountered as other colonic pathologies like DD may also have a similar presentation, reinforcing the concept of maintaining a high degree of suspicion [35].

RCC may present with iron deficiency anaemia and weight loss, with these non-specific changes having implications for delayed diagnosis [36]. Tumour biology of RCC can sometimes make them aggressive due to the high prevalence of microsatellite instability (MSI) and CpG island methylator phenotypes (CIMP) [37].

This stresses the importance of thorough colonic evaluation after an acute episode of diverticulitis [38, 39]. The presence of right-sided and transverse colon tumours means that colonoscopic evaluation following an attack of acute diverticulitis is required [40, 41].

### Challenges in follow-up practices

Approximately half (53%) of our patients underwent colonic evaluation within a year of their acute diverticulitis episode divided equally between those having a colonoscopy versus flexible sigmoidoscopy. This practice reflects the inconsistencies in follow-up care and the lack of structured and standardised guidelines.

ACPGBI guidelines (2022) recommend routine colonoscopy or radiological investigations for patients with complicated diverticulitis only, or in those with suspicious features on CT scan (thickening, mass), or in the presence of other risk factors. Uncomplicated diverticulitis cases are excluded from this recommendation [13, 14].

The American Society of Colon and Rectal Surgeons (ASCRS) recommends full colonic assessment in instances of complicated diverticulitis but for uncomplicated disease, the practice remains more selective and tailored according to clinical evaluation and risk stratification [40, 41].

We demonstrated multiple strongly associated factors with a diagnosis of CRC, including Hinchey grade 1b, elevated CRP, young age, and male gender. Therefore, both of these guidelines may potentially be excluding high-risk patients that would otherwise benefit from luminal investigations.

Both ACPGBI and ASCRS highlighted the role of CT scans in identifying suspicious lesions needing further assessment. These may be missed during routine CT reporting and mis-diagnosed as inflammation from acute diverticulitis. The review of CT scans by specialist gastrointestinal radiologist will help improve the quality of detecting early or malignancy-associated features. This targeted approach as per the ACPGBI’s recommendations to perform colonoscopies in patients with suspicious CT findings will allow better service utilisation and the avoidance of un-necessary procedures.

Elderly patients (above the age of 80 years) were less likely to be followed up with colonic investigation (OR 0.53, *P* = 0.016) in our findings. This could be related to concerns regarding compliance with bowel cleansing agents, associated comorbidities, and the inherent risks of endoscopic procedures. Therefore, age-specific guidelines and protocols are required to cater for this particular demographic [42].

Furthermore, the choice between flexible sigmoidoscopy and colonoscopy varies according to available resources and clinician decision. Colonoscopy provides a comprehensive evaluation but may not always be available/possible, indicated, or necessary [43]. Flexible sigmoidoscopy and CTC may be alternative options in elderly patients or in those with anticipated difficult colonic anatomy [44].

### Implications for practice and research

Despite the interest in DD [45], there is variability in management protocols globally. Frequently, healthcare systems are limited by resource availability and socio-economic status. In certain geographical locations (Asian countries), a more cautious approach is taken favouring routine endoscopic evaluation of the colon following diverticulitis irrespective of disease severity [46]. Interestingly, four out of ten of our cancer cases were classified as Hinchey 1a.

Advanced imaging modalities such as enhanced CT, magnetic resonance imaging (MRI), or positron emission tomography (PET) scans may help to improve diagnostic accuracy. Moreover, a tailored follow-up approach may help in better use of limited resources.

However, defining population groups most at risk is needed in future research allowing standardised guidelines to be established [32]. Colonoscopy should be regarded as the ‘gold standard’ in evaluating patients with acute diverticulitis (due to findings of more proximal lesions) but a consensus is required especially in patients with equivocal CT findings [43, 44].

### Limitations

This study is not without its limitations. Firstly, due to its retrospective design, it lends itself to inherent biases including the potential to introduce selection bias as only patients with CT-confirmed diverticulitis were included. Secondly, the follow-up period of 12 months may under-represent/mis-represent the true incidence of CRC occurrence. Thirdly, our study was multicentre, allowing for differences in clinical practice and follow-up plans. However, this may increase the power of our findings and make the conclusions more reproducible and generalisable. Finally, there may have been discrepancies between the number of patients offered follow-up investigations versus the number that actually attended. Our analysis is applicable to the demographics and diversity of the UK population and therefore may not apply in other patient cohorts.

## Conclusion

Our finding of the incidence of CRC in patients presenting with acute diverticulitis is in line with published literature. Mild diverticulitis (Hinchey grade Ib) cases in particular may need further assessment and their CT findings reviewed by a specialist radiologist. There is a need for uniform, consistent, standardised care protocols and follow-up pathways to optimise care in patients with acute diverticulitis and avoid missing malignant lesions. Additionally, further guidance on select high-risk groups is required.

## Supplementary Information

Below is the link to the electronic supplementary material.

Supplementary file1 (DOC 81 KB)

## Data Availability

No datasets were generated or analysed during the current study.
